# The relationship of non-cognitive factors to academic and clinical performance in graduate rehabilitation science students in the United States: a systematic review

**DOI:** 10.3352/jeehp.2021.18.31

**Published:** 2021-11-23

**Authors:** Kelly Reynolds, Caroline Bazemore, Cannon Hanebuth, Steph Hendren, Maggie Horn

**Affiliations:** 1 Physical Therapy Division, Duke University School of Medicine, Durham, NC, USA; 2 Rocky Mountain University of Health Professions, Provo, UT, USA; 3 Duke University Medical Center Library, Duke University, Durham, NC, USA; Hallym University, Korea

**Keywords:** Academic performance, Achievement, Emotional intelligence, Self-efficacy, Students

## Abstract

**Purpose:**

Rehabilitation science programs utilize cognitive and non-cognitive factors to select students who can complete the didactic and clinical portions of the program and pass the licensure exam. Cognitive factors such a prior grade point average and standardized test scores are known to be predictive of academic performance, but the relationship of non-cognitive factors and performance is less clear. The purpose of this systematic review was to explore the relationship of non-cognitive factors to academic and clinical performance in rehabilitation science programs.

**Methods:**

A search of 7 databases was conducted using the following eligibility criteria: graduate programs in physical therapy (PT), occupational therapy, speech-language pathology, United States-based programs, measurement of at least 1 non-cognitive factor, measurement of academic and/or clinical performance, and quantitative reporting of results. Articles were screened by title, abstract, and full text, and data were extracted.

**Results:**

After the comprehensive screening, 21 articles were included in the review. Seventy-six percent of studies occurred in PT students. Grit, self-efficacy, emotional intelligence, and stress were the most commonly studied factors. Only self-efficacy, emotional intelligence, and personality traits were examined in clinical and academic contexts. The results were mixed for all non-cognitive factors. Higher grit and self-efficacy tended to be associated with better performance, while stress was generally associated with worse outcomes.

**Conclusion:**

No single non-cognitive factor was consistently related to clinical or academic performance in rehabilitation science students. There is insufficient evidence currently to recommend the evaluation of a specific non-cognitive factor for admissions decisions.

## Introduction

### Background/rationale

In rehabilitation science programs, traditionally cognitive factors such as grade point average (GPA) and standardized test scores, have been heavily weighted in admissions decisions [1] and used for matriculated students to predict academic performance in the didactic curriculum and licensure exam scores [2]. While these cognitive variables are generally considered good indicators of future academic performance, most studies show that they explain less than half of the variance in academic outcomes for speech-language pathology (SLP) [3] and physical therapy (PT) [4] students. Years of psychology research demonstrate that intelligence alone is not the sole predictor of academic performance and often not even the strongest [5]. Students with equivalent intelligence can exhibit highly variable academic achievement. To better understand and explain this variability, extensive investigation into the relationship of non-cognitive factors to academic performance has been explored in many student populations [6]. Non-cognitive factors are especially relevant to graduate rehabilitation science students that must demonstrate proficiency across the cognitive, psychomotor and affective learning domains in both the classroom and clinical settings to graduate and be eligible for licensure.

Graduate rehabilitation science programs in PT, occupational therapy (OT), and SLP include didactic and clinical components with formal evaluation in all 3 learning domains. While most written assessments in these programs evaluate the cognitive domain, practical examinations test students’ psychomotor skills and clinical experiences require them to merge their cognitive knowledge and psychomotor skills with the affective domain. It is generally agreed upon that to be successful in the classroom and clinical environments, rehabilitation therapists should possess many non-cognitive factors such as strong interpersonal and communication skills, collaborative spirit, ethical decision-making, and empathy [7]. However, these factors are inconsistently evaluated before or after matriculation and their relationship to performance is unclear. A review of 5 non-cognitive factors influence on academic performance in health professions students found inconsistent results among studies, and the authors caution against broad interpretation and implementation until more is known [8]. Our review aimed to expand upon this by examining the rehabilitation professional student population specifically, which is unique from medical, dental, and other health professions students. We also evaluated both academic and clinical performance metrics to elucidate the impact of non-cognitive factors in the classsroom and clinical environments and did not predetermine which non-cognitive variables to include.

Although there is a burgeoning interest in evaluating non-cognitive attributes in PT, OT, and SLP applicants and developing these in matriculated students, there is currently no consensus on which non-cognitive factors should be examined. This is partially because “non-cognitive” has been used as an all-encompassing term in the literature to include any factor outside of GPA and standardized test scores [9]. Some authors consider interviews and/or letters of recommendation under the non-cognitive umbrella [10]; others include constructs such as critical thinking, clinical reasoning, or reflection [2]. This broad definition presents a great challenge when attempting to synthesize the literature and draw conclusions. For this review, the authors utilized a comprehensive framework presented by Richardson et al. [5] that describes 5 non-intellective constructs to narrow the non-cognitive definition. The 5 dimensions they posit are personality traits, motivational factors, self-regulatory learning strategies, students’ approaches to learning, and psychosocial contextual factors [5]. These non-intellective constructs were used to guide the selection criteria for this review.

### Objectives

Therefore, the aim of this systematic review was to examine the relationship of non-cognitive factors and metrics of success in academic and clinical performance in graduate rehabilitation therapy professional students to inform admissions practices and enhance student support.

## Methods

### Ethics statement

This review utilized previously conducted work and did not require institutional review board approval.

### Study design

This systematic review was developed following the Preferred Reporting Items for Systematic Reviews and Meta-Analyses 2020 statement. It includes a 27-item checklist and flow diagram outlining best-practice reporting for systematic reviews [11].

### Eligibility criteria

The selection criteria were determined for the population, the study intervention, the reported outcomes, and the publication types. Studies were included if their participants were students enrolled in graduate-level studies for PT, OT, or SLP. The study intervention criteria included assessments of non-cognitive factors such as grit, self-efficacy, or resilience. Specifically, studies assessing the students for non-cognitive factors within the domains set forth by Richardson et al. [5] were included. Additionally, studies that reported outcomes of either licensure exam results, graduate coursework GPA, grades, or clinical performance were included. Only studies conducted in the United States were included as there is considerable heterogeneity in both educational and practice standards for rehabilitation science professionals globally. Publication types included were peer-reviewed journal articles and doctoral dissertations.

Studies were excluded if they: (1) included wrong populations, such as physical therapy assistant students, undergraduate students, or other health professions such as medical, dental or nursing; (2) examined cognitive factors such as undergraduate GPA or standardized test scores, or included variables that could be construed as rooted in cognition (e.g., critical thinking, metacognition); (3) did not report non-cognitive factors; (4) utilized admissions criteria assessments as outcome measures; or (5) were conducted outside the United States.

### Information sources

A comprehensive systematic search of the literature was conducted using PubMed, CINAHL, Embase, Scopus, ERIC, Education Database, and MedEdPortal databases.

### Search strategy

The original search strategy was executed on July 6, 2020, and updated on August 19, 2021 and studies published up until this date were included. The literature search plan was performed in collaboration with a research librarian (S.H.). An additional hand-search of the literature was conducted to ensure the inclusion of additional appropriate studies meeting study criteria.

### Selection process

Once the literature search query was completed, the resulting studies were uploaded to Covidence (Melbourne, Australia) [12] to be consolidated and organized for review (S.H.). Following an initial removal of duplicate findings, the abstract and title screening was conducted by 2 independent review authors (C.B., C.H.) to identify studies meeting inclusion criteria. Any disagreements were resolved by a third review author (K.R.). The full texts were further independently assessed for eligibility by 2 review authors (K.R., C.B.), with any disagreements resolved in discussion with a third review author (M.H.).

### Data collection process

A standardized, pre-piloted form was used to extract the data from the included studies.

### Data items

Extracted information included (1) information on the population including the type of study, student time in the program, number of time points measured, and number of students; (2) the type of non-cognitive factor assessed, and the assessment tool utilized to measure it; (3) the outcome measure selected, and any secondary outcomes included; and (4) statistical results measuring the relationships between the non-cognitive factors assessed and the outcome measures selected. One review author extracted the data independently (C.B.), with discrepancies resolved and a review conducted by a second review author (K.R.).

### Study risk of bias assessment

The McMaster Critical Review Form for Quantitative Studies (MCRF) was utilized to assess the quality of included studies and the risk of bias. It has been established as a reliable tool for critical appraisal [13]. A modified version of the MCRF that has been previously utilized to review educational literature was used in this review and included 11 criteria instead of the standard 10 [2]. Two review authors (K.R., C.H.) independently assessed the risk of bias for each included study, scoring items as a 0 if the criterion was not met or absent and a 1 if the criterion was met or present. Disagreements were resolved by a third author (M.H.) when necessary.

### Effect measures

Outcomes were reported as correlation coefficients (Pearson’s product-moment correlation [r] or Spearman’s rho [r_s_]) and/or goodness of fit measures (R^2^) for regression models. The magnitude of the effect was determined following Cohen’s established criteria of 0.1 to 0.3 indicating a small association, 0.3 to 0.5 indicating a moderate association, and 0.5 to 1.0 indicating a large association [14].

### Synthesis methods

We categorized the outcomes by setting, dividing them into clinical and academic. Comparable non-cognitive variables as named by the original study authors (e.g., emotional intelligence) were grouped for analysis and the presentation of results (Dataset 1). Some studies evaluated more than 1 non-cognitive factor and/or performance in more than 1 setting. The results of those studies were considered and reported separately for each factor and each setting.

### Reporting bias assessment

To minimize outcome reporting bias, the authors established specific outcome criteria before performing the literature search. No core outcome set was available for the review topic. The grey literature comprising conference abstracts and doctoral dissertations were included in the search and doctoral dissertations were included in the review because they are considered peer-reviewed by the dissertation committee. Doctoral dissertation results that were not published as peer-reviewed articles were retained in the review to minimize outcome reporting bias.

### Certainty assessment

All data included in the review were taken from level 3 observational studies including case-control study, retrospective comparative study, which typically lowers the initial level of certainty. However, this is an appropriate study design for the research question which calls for correlational or predictive statistical analyses to examine relationships among independent and dependent variables. Certainty is further complicated by small sample sizes and frequent consideration of students from a single program in most included studies which are addressed under limitations. The best available evidence was included in this review, but suggestions for future research to improve the certainty of findings are discussed below. Review authors performed a risk of bias assessment using the MCRF for each study as well as a reporting bias assessment to improve the certainty of findings.

## Results

### Study selection

**Fig. 1.**
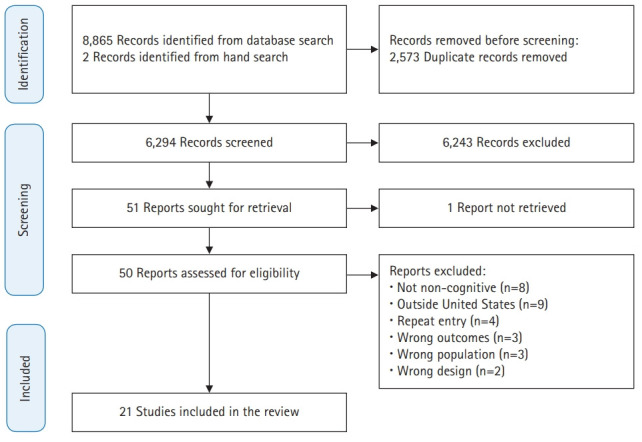
Preferred Reporting Items for Systematic Review and Meta-Analyses diagram.

A total of 8,865 studies were identified using an electronic database search and 2 additional studies were identified via hand search. After 2,573 total duplicates were removed, title and abstract screening yielded 51 studies eligible for full-text review. Twenty-one articles met the eligibility requirements for the review (Fig. 1).

### Study characteristics

The 21 studies identified for full-text review included 2,843 students across 3 rehabilitation science domains (PT, OT, SLP). All studies included in the review were observational, level 3 studies published between 2002 and 2021. Fifteen studies explored non-cognitive factors in PT students (n=2,254) [15-29], 3 studies evaluated OT students (n=328) [30-32], 2 studies examined SLP students (n=108) [33,34], and 1 study included a mixed cohort of PT and OT students (n=153) [35]. Six of the 21 studies evaluated non-cognitive factors as they related to clinical performance, while 13 articles evaluated the relationship of non-cognitive factors to academic performance. Only 2 studies evaluated the relationship of non-cognitive factors to both clinical and academic performance [16,24]. More than 10 unique non-cognitive characteristics were reported across the studies with variable relationships with clinical and academic performance (Dataset 1). Grit, self-efficacy, emotional intelligence, and stress were the most commonly studied factors.

### Risk of bias in studies

The results for the modified McMaster Critical Review Form for Quantitative Studies are shown in Table 1. The quality scores ranged from 7 to 10 out of 11, with a mean of 8.90.

### Results of individual studies

Individual summary statistics and conclusions for each study included in the review are presented in Dataset 1.

### Results of syntheses

#### Clinical performance

Emotional intelligence and self-efficacy were the only factors evaluated in more than 1 study examining the relationship to clinical performance. The role of emotional intelligence remains unclear as results from 4 studies were conflicting, demonstrating both positive and inverse relationships as well as no relationship. Overall, emotional intelligence is not a strong predictor of clinical performance. Results for self-efficacy were also mixed; in 2 of 3 studies, students with higher self-efficacy demonstrated stronger clinical performance. Articles evaluating non-cognitive factors and clinical performance included students from all 3 rehabilitation science programs while academic performance was almost exclusively limited to PT.

#### Academic performance

Emotional intelligence, grit, and stress were evaluated in multiple studies examining the relationship to academic performance. Both articles evaluating emotional intelligence found that it was unrelated to GPA. Grit was examined in 4 included studies. The majority found that grit had moderate, positive, significant correlations with academic performance, the strongest of all relationships examined in this review. Generally, students that rate themselves as grittier have higher program GPAs. Stress was assessed in 4 studies and demonstrated inconsistent results. Two studies found that higher levels of stress were weakly, but significantly related to lower GPA while 2 additional studies found no relationship. Of note, all 4 studies of stress used different scales to quantify stress.

### Reporting biases

All studies except 1 reported their statistical results for the outcomes considered in this review. Velis et al. [32] noted that they found no relationship between OT students’ learning style and their clinical performance. However, the specific statistical findings for this analysis were not reported, thus we are unable to verify their conclusions. Authors did note differences among learning styles and 1 subcategory of clinical performance (management) with thorough statistical reporting of this analysis and several others beyond the scores of this review so this study was retained.

### Certainty of evidence

There is low certainty of the evidence for all non-cognitive factors examined and their relationship to clinical and academic performance. The low certainty occurred for some reasons: (1) small sample sizes in most studies with only 2 studies including power analyses [19,26], (2) heterogeneity in findings among studies, and (3) observational study design.

## Discussion

### Key results

The purpose of this systematic review was to evaluate the relationship of non-cognitive factors and clinical and academic performance in graduate rehabilitation science students. We performed a synthesized evidence appraisal and determined grit is the non-cognitive factor most linked to academic success while stress is occasionally related to worse academic outcomes. Greater self-efficacy was related to better clinical performance in studies of PT and SLP students, but not OT students. All other non-cognitive factors were either present in only a single study, demonstrated variable results, or had no relationship to performance, limiting our ability to draw definitive conclusions.

### Interpretation

#### Non-cognitive factors and clinical and academic performance

Few non-cognitive factors were studied across clinical and classroom environments, despite the importance of rehabilitation science students demonstrating proficiency in both arenas. Emotional intelligence, self-efficacy, and personality traits were the only factors evaluated in both contexts. Vandenberg found that emotional intelligence was inversely related to clinical performance in PT students during their final clinical rotation, but unrelated to academic performance in the same subjects [24]. This finding indicates that students with lower emotional intelligence scores perform better clinically which she notes is an unexpected and concerning result [24]. Utsey found that higher self-efficacy was correlated with better clinical and academic performance in a sample of PT students [16]. Personality traits were not found to predict clinical success in SLP students [34] nor first time licensure exam pass rates in PT students [23].

Success is evaluated via different metrics in the clinical and academic environments and the factors required to excel in each may or may not overlap. Many traits such as grit, empathy, and a collaborative spirit are considered desirable in rehabilitation professionals [7,36], but are unlikely to be directly evaluated during the didactic portion of the rehabilitation sciences curriculum. If these factors are not formally assessed, research may not exhibit a relationship to academic performance despite their importance. To tease out if specific factors are more relevant in one environment, studies of non-cognitive factors must evaluate both academic and clinical performance. There is a paucity of evidence on non-cognitive factors spanning both contexts which decrease the utility of these factors as predictors of performance. Future research should aim to evaluate non-cognitive factors and the relationship to both academic and clinical performance in a single sample to provide deeper insight for programmatic use.

#### Non-cognitive factors and clinical performance

Emotional intelligence, self-efficacy, learning styles, and personality traits were studied in relation to clinical performance. Students’ self-reports of emotional intelligence and personality traits were not independently related to clinical performance in rehabilitation science students. Interestingly, while students’ ratings of their emotional intelligence did not positively correlate to clinical performance when clinical instructors (CIs) were asked to rate their students’ emotional intelligence, the CIs’ scores demonstrated moderate, positive, and significant correlations with clinical performance scores [30]. This indicates that emotional intelligence may be a relevant skill for successful clinical care; however, students may be inaccurate raters of their emotional intelligence, typically rating themselves higher than their CIs. Three unique instruments were used to rate emotional intelligence and study authors from one study speculated that the instrument may not capture the type of emotional intelligence needed to excel clinically [15]. Another study evaluated 4 learning styles using Kolb’s Learning Styles Inventory and found that students who prefer active experimentation are stronger clinically than those who prefer reflective observation [32]. Active experimenters favor experiential learning through trial and error; reflective observers first watch skilled clinicians and cognitively process the experience before attempting a skill. Students with an active experimentation learning style could be perceived by CIs as more engaged and participatory in patient care and subsequently score higher on clinical performance metrics [32]. Learning styles and clinical performance were only evaluated in a single study, limiting the generalizability of these findings. Self-efficacy was examined in 3 studies and was the only non-cognitive variable evaluated in all 3 rehabilitation science disciplines. It was found to have a small to moderate correlation with clinical performance in PT and SLP students but was unrelated to clinical performance in OT students. The mixed results may be due to the measures used. Three separate tools were used to quantify self-efficacy and 3 different tools were used to measure clinical performance leading to a highly variable study methodology. Ultimately, self-efficacy may have the most utility when considering students’ potential clinical performance. No other variable emerged as a consistent predictor of clinical performance.

#### Non-cognitive factors and academic performance

Emotional intelligence, grit, psychosocial factors (anxiety, stress, depression), confidence, empathy, self-efficacy, learning strategies, personality traits, coping skills, and multiple domains from the Non-Cognitive Questionnaire-revised (NCQ-R) were studied in relation to academic performance, typically measured by GPA, licensure exam scores, or pass rate. All studies of academic performance included PT students and 1 of the 16 studies included a mixed cohort of PT and OT students. No studies included SLP students. Emotional intelligence, confidence, learning strategies, personality traits, and coping skills were unrelated to academic performance despite prior research in other health professions students that have demonstrated the Big Five personality trait of conscientiousness to positively predict academic success [8]. Although the NCQ-R total score did not predict licensure exam scores, select components (long-range goals, leadership, community ties, and academic familiarity) taken collectively were significant predictors [22]. This suggests that subcomponents of some existing surveys may have better practical utility in informing students’ potential performance and careful analyses must be conducted to reveal relevant relationships. Psychosocial factors demonstrated mixed results on academic performance. Past research in different student populations has generally supported the idea that stress, anxiety, and depression negatively impact students’ mental health leading to worsening cognitive function [5,37,38]. This was supported by data showing small, but significant inverse relationships between stress, anxiety, and depression and GPA in a sample of over 1,200 PT students [19] and a second smaller study [29]. Two additional studies did not find the same relationship, possibly because their samples were drawn from individual programs where students’ stress and anxiety may be mediated by program influences such as pace or the associated learning environment. Empathy was evaluated in a single study and was highly correlated to practical examination scores. Authors note that the practical examination rubric specifically addressed and allotted points for “personal interactions including addressing the patient appropriately and politely, building rapport, paying attention, actively listening, demonstrating appropriate body language, and utilizing empathy” [26]. This study of empathy highlights the previously addressed notion that some desirable professional traits may need to be included in academic evaluation metrics to reveal a relationship with academic performance [26]. Practical examinations are common in the health professions and offer an opportunity to focus on and potentially develop desirable non-cognitive factors. Self-efficacy was found to have a small to a moderate relationship with academic performance and was a significant predictor of GPA. Much like clinical performance, students that indicated higher self-efficacy scores demonstrated stronger academic performance throughout PT school [16]. Students with higher levels of self-efficacy spend more time studying, engaging with complex material, and persevering in their academic pursuits than students with lower self-efficacy, likely contributing to their superior performance [39]. Grit, defined as “passion and perseverance towards long-term goals” [40] has received a great deal of attention in the literature in recent years as a non-cognitive factor capable of distinguishing those who will succeed and excel based on their inherent commitment to a goal. Grit demonstrated the strongest relationship to the academic performance of all non-cognitive factors included in this review; however, most authors caution against using grit in admissions decisions until further research is completed [17,40].

### Limitations

This systematic review presents some limitations. All of the included articles were rated as level 3 evidence, which is not surprising given the research question which warrants an observational design. Several of the studies include small sample sizes from a single rehabilitation science program, limiting their external validity. No single non-cognitive factor was evaluated in more than 4 studies and several have only been studied once, making it difficult to draw broad conclusions, especially around which non-cognitive factors to include in major decisions such as program admissions. Finally, the timing of data collection may impact the findings. Most included studies are cross-sectional versus longitudinal in design. It is not well understood if various non-cognitive factors change throughout graduate rehabilitation sciences curriculum. We must exercise caution in using non-cognitive variables collected at later points in the curriculum for purposes such as admissions without knowing if these variables are static or mutable.

### Suggestions

The majority of studies evaluate a single non-cognitive factor and the relationship to one metric of performance, either academic or clinical. Future studies should build upon the existing work and consider evaluating several non-cognitive variables simultaneously to develop a multivariate prediction model for greater practical utility. Future research should also endeavor to evaluate the relationship of these noncognitive variables on both academic and clinical performance to create a more holistic picture of their influence. All but one study in this review limited their populations to a single rehabilitation discipline. Interprofessional collaboration in future non-cognitive studies will provide more robust data and larger sample sizes. This review revealed that a wide array of measurement tools have been used thus far to quantify various non-cognitive factors and metrics of performance. Going forward, researchers should make use of previously employed measurement tools to allow for stronger comparisons between studies and reduce confounding factors.

## Conclusion

The results of this review provide guidance for educators as well as future researchers interested in non-cognitive factors. While grit, self-efficacy, and stress present a promising start into developing a deeper understanding of the impact of non-cognitive factors on performance, there is insufficient data at present to encourage the use of these factors exclusively in admissions decisions or to determine which students may need additional academic or clinical support in rehabilitation sciences programs.

## Figures and Tables

**Table 1.** Risk of bias assessment via Modified McMaster Critical Review Form for Quantitative Studies

Study	Level of evidence	Total score	Critical appraisal category										
			1	2	3	4	5	6	7	8	9	10	11
Gordon-Handler[30] (2010)	3	8	1	1	0	0	1	1	1	1	1	0	1
Lewis[15] (2010)	3	9	1	1	1	0	1	1	1	1	1	0	1
Andonian[31] (2013)	3	9	1	1	1	0	1	1	1	1	1	0	1
Vandenberg[24] (2019)	3	8	1	1	0	0	1	1	1	1	1	0	1
Pasupathy et al. [33] (2013)	3	7	1	1	0	0	0	1	1	1	1	0	1
Utsey[16] (2006)	3	9	1	1	1	0	1	1	1	1	1	0	1
Velis et al. [32] (2015)	3	9	1	1	1	0	1	1	1	1	1	0	1
Richardson et al. [34] (2020)	3	9	1	1	1	0	1	1	1	1	1	0	1
Huhn et al. [27] (2021)	3	9	1	1	1	0	1	1	1	1	1	0	1
Carp et al. [17] (2020)	3	10	1	1	1	0	1	1	1	1	1	1	1
Bliss et al. [18] (2020)	3	9	1	1	1	0	1	1	1	1	1	0	1
Richardson et al. [25] (2020)	3	9	1	1	1	0	1	1	1	1	1	0	1
Bogardus [19] (2019)	3	10	1	1	1	1	1	1	1	1	1	0	1
Frank et al. [20] (2005)	3	10	1	1	1	0	1	1	1	1	1	1	1
Flowers et al. [29] (2020)	3	8	1	1	0	0	1	1	1	1	1	0	1
Douris et al. [28] (2021)	3	10	1	1	1	0	1	1	1	1	1	1	1
Alexander et al. [21] (2016)	3	8	1	1	1	0	0	1	1	1	1	0	1
Richardson et al. [26] (2021)	3	10	1	1	1	1	1	1	1	1	1	0	1
Pucillo et al. [35] (2021)	3	9	1	1	1	0	1	1	1	1	1	0	1
Guffey et al. [22] (2002)	3	9	1	1	1	0	1	1	1	1	1	0	1
Galleher et al. [23] (2012)	3	8	1	1	0	0	1	1	1	1	1	0	1

Level of evidence: 3=non-experimental, correlational, and/or cohort study. Critical appraisal category scoring key: 1=study purpose stated clearly; 2=relevant literature reviewed; 3=sample described in detail; 4=sample size justified; 5=outcome measures reliable; 6=outcome measures valid; 7=results reported in terms of statistical significance; 8=analysis methods appropriate; 9=educational importance reported; 10=dropouts reported; 11=conclusions appropriate.

## Footnotes

**Data availability**
Data files are available from Harvard Dataverse: https://doi.org/10.7910/DVN/B3Q9RN Dataset 1. Individual summary statistics and conclusions for each study.

## Supplementary materials

Supplement 1. Audio recording of the abstract.
